# A review of microcredentials in health professions continuing professional development

**DOI:** 10.3389/fmed.2025.1532811

**Published:** 2025-02-03

**Authors:** Kelly Womack-Adams, Kathryn A. Morbitzer, Christine Ondek, Heidi Collins, Jacqueline E. McLaughlin

**Affiliations:** University of North Carolina Eshelman School of Pharmacy, Chapel Hill, NC, United States

**Keywords:** microcredential, digital badge, microcertification, health professions, continuing professional development

## Abstract

Microcredentials are an emergent tool to support knowledge and skill development. Despite their growing popularity in medical education – and higher education more broadly – it is unclear how these strategies have been utilized to support continuing professional development in the health professions. A rapid systematic review was conducted to explore the current relevant literature due to the timely and evolving nature of microcredentials. PubMed, Embase, and ERIC were used for the article search. Of the 290 relevant articles found from the searches, a total of 11 articles were included after abstract and full-text screenings. All articles used in this review were published within the past 10 years. Microcredentials were used across various professions, covered a wide range of topics, and employed various teaching strategies. The definitions used for key terms like microcredential were inconsistent across articles.

## 1 Introduction

Microcredentials – including digital badges, micro-learning with certifications, and micro-certifications – are an emerging strategy for incentivizing and verifying knowledge and skill acquisition across various disciplines. Despite their growing popularity in medical education – and higher education more broadly – it is unclear how these strategies have been specifically utilized to support continuing professional development in the health professions (1–3).

Microcredentials are typically awarded after completion of short courses or training modules to indicate that an individual has demonstrated mastery of a specific skill or topic (1, 3, 4). The terms microcredential, digital badge, and microcertification may be used interchangeably to represent a learning experience that is significantly shorter than a traditional academic degree or license; for simplicity and consistency, the term microcredential will be used in this paper moving forward (3). Micro-learning is a microcredential learning structure involving smaller units of learning such as questions with explained solutions or brief modules for learning specific content (5).

Scholars from various disciplines have described the use of microcredentials, including education, welding, and engineering (6–8). Research touts microcredentials for personalized professional development that individuals can use to more accurately demonstrate skills and competencies to employers (3, 6, 7). Due to the personalized nature of microcredentials, there is often substantial variety between microcredentials. Microcredentials are also frequently used to reskill and upskill, including learning new concepts (9). Some disciplines are also implementing them as supplementary to traditional degree pathways, helping candidates differentiate themselves more effectively with potential employers (7, 10).

Challenges with microcredentials include inconsistent terminology, varied credential goals and outcomes, as well as low awareness of this approach to workforce development (3, 9, 10). Variation within different microcredentials and the importance of each individual certification of microcredentials also varies significantly (3).

Despite these challenges, there is a prominent view that microcredentials are beneficial, particularly in post-pandemic learning (3, 11). The literature on microcredentials in higher education increased notably after 2020, representing a rapid increase in interest post-pandemic (11). This may be because the pandemic led to many career shifts, with employees needing certifications to indicate their skills, and accessible learning for them (11). The short course nature of microcredentials made flexible and timely professional development feasible, especially when offered through virtual platforms.

As an emerging topic, there is still much to be learned about the use of microcredentials, especially for specific fields like health professionals. The purpose of this study was to review how microcredentials have been utilized to support the development of health professionals and more broadly understand how they might be integrated into continuing professional development (CPD). Ultimately, this work will provide an evidence-based foundation upon which CPD programs can build microcredentialing systems that address current and emerging challenges to knowledge and skill acquisition among healthcare providers.

## 2 Methods

Due to the current and rapidly evolving nature of microcredentials, a rapid review was utilized for this study. A rapid review involves the same rigorous methodology as a systematic review but is completed on a condensed timeline, an average of 3.2 months compared to typically a year or more for a systematic review (12). This methodology is beneficial for timely questions that require quicker answers.

### 2.1 Search terms and databases

To better understand microcredentials in the health professions, the following search terms were used:

(micro-cred* OR microcredential* OR micro-cert* OR microcertification* OR "digital badge" OR "digital badges" OR micro-learning* OR microlearning) AND (health education OR health profession* OR healthcare OR medicine OR medical OR doctor OR physician OR pharmacy OR pharmacist OR nurse OR nursing OR dentistry OR dental OR dentist

The search terms and their variations helped ensure we were finding as many articles as possible related to microcredentials in healthcare CPD. The search was conducted in September 2024 using PubMed, EMBASE, and ERIC to allow for a breadth of possible articles related to microcredentials in healthcare CPD. ERIC, for example, is a database for all research and journal articles related education in any capacity, including medical education. The references for included articles were also reviewed by hand to source additional relevant articles that may have been published in unindexed journals.

### 2.2 Article evaluation and inclusion criteria

All search results were uploaded to Covidence and duplicates were removed. Title and abstract screening was conducted independently by two researchers (KWA and CO). Disagreements were settled through discussion until consensus was reached. The same two researchers conducted full text review. There was 94% agreement for the full text review and the two researchers met to discuss any disagreements until full consensus was reached.

Articles were included that:

1. Described a microcredential, digital badge, microcertification, or microlearning that provided credit designed for continuing education or professional development.
2. Included healthcare professionals as learners.
3. Were empirical studies, including qualitative, quantitative, and mixed-methods research that focused on outcomes or perspectives of one of the types of continuing education listed in inclusion criteria [1].
4. If the article discussed a specific microcredential, to be included in this review, it had to also include discussion after implementation, i.e., evaluations or reflections.
5. Were published in English.

Articles were excluded that:

1. Focused on health professions education (e.g., graduate, postgraduate, or undergraduate programs).
2. Did not focus on health professionals’ learning.
3. Focused on traditional certifications, degrees, or other forms of credentialing not related to microcredentials, digital badges, microcertifications, or continuing education microlearning that provided credit.
4. Were opinion pieces, editorials, books, dissertation and theses, literature reviews, conference abstracts, and non-empirical articles.

A key focus for this review was on what is currently understood about the effectiveness and implementation of microcredentials in CPD. This meant that articles that focused only on the design or preparation of a specific CPD were not included.

### 2.3 Data extraction

A data extraction tool was developed and used in Excel. The codes in the tool were developed *a priori* and were guided by the codebook developed by Noyes and colleagues in their review of digital badges (13). The codebook used in this rapid review can be seen in the Supplementary material. Text-based data was copied and pasted into Excel from the articles for analysis.

Two researchers (KWA and JM) independently extracted data from 3 articles. Given high agreement between researchers (96% agreement), and following consensus building, the remaining data was extracted by one researcher (KWA). Deductive thematic analysis based on the *a priori* codes was used to find patterns in the coded data. Findings are represented with frequency and percentage.

## 3 Findings

**Figure 1:**
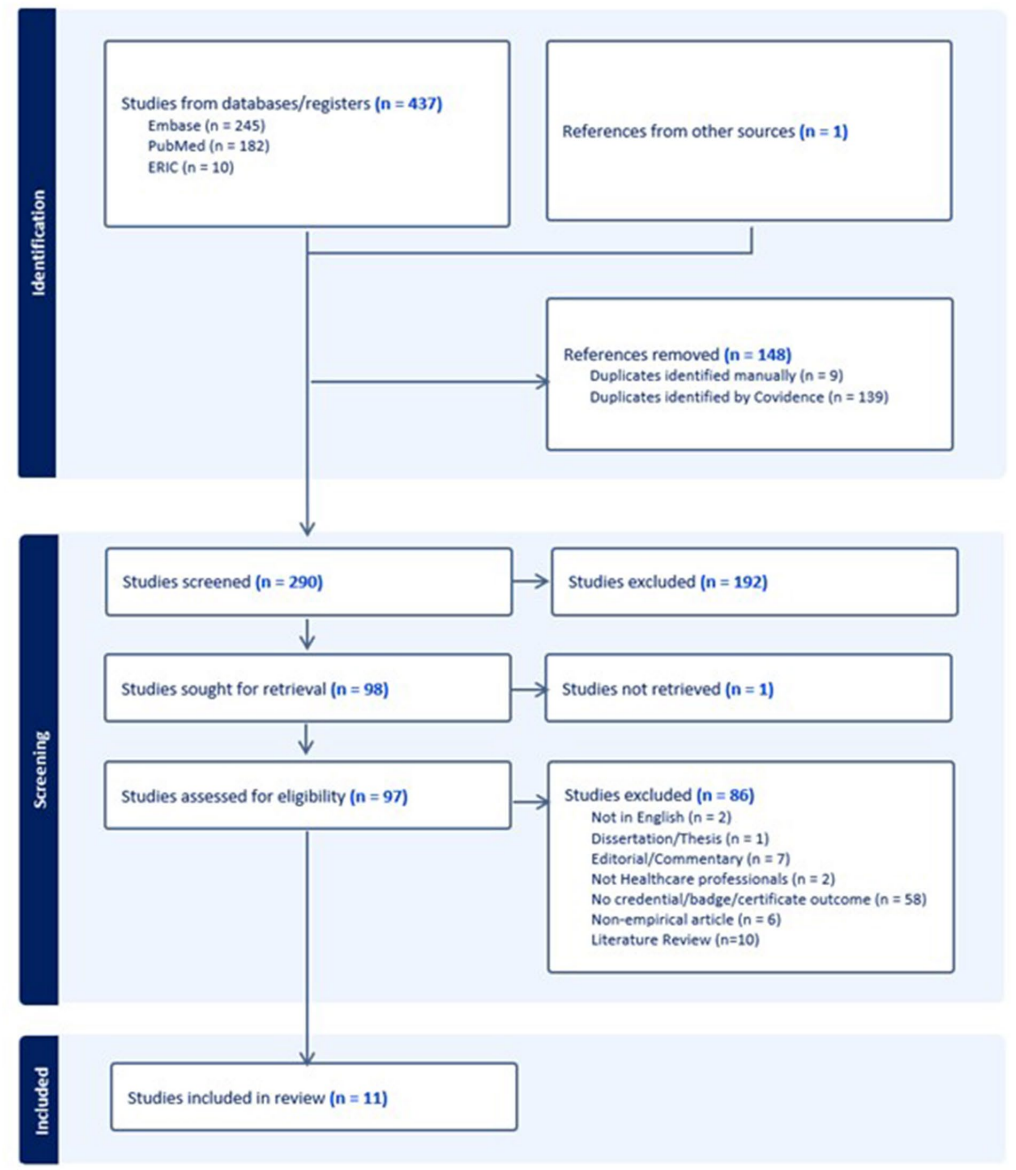
PRISMA diagram for literature review on microcredentials in health professions continuing education.

From the search terms, 182 articles were found on PubMed, 245 on EMBASE, and 10 from ERIC. One article was identified through our references search by hand and included in the review. A total of 148 duplicates were removed, leaving 290 articles for evaluation. Of those found, 192 were deemed irrelevant during the title and abstract screening. After full text evaluation, 86 were excluded and one article was not able to be obtained, which left 11 articles for extraction (Table 1). The search strategy is summarized in the PRISMA diagram in Figure 1. Summary of the findings are listed in Table 2.

**Table 1:** List of articles included in this rapid review.

Citation	Focused type of credential*	Focused Healthcare Discipline
Goodenough et al. (2020) (20)	Certificate for Professional Education Credits	Nurses, Personal Care Assistants
Bobbitt et al. (2023) (18)	CPD Credits for Microlearning	Nurses, Pharmacists, Physicians
Romero-Clara et al. (2024) (19)	Professional Credits for Microlearning	Nurses, Physicians, Researchers, Pharmacists
Rohan et al. (2017) (22)	Digital Badge	Nurses
DeMarco et al. (2024) (15)	Digital Badge	Clinical Research Coordinators
Chang et al. (2019) (14)	Digital Badge	Physicians, Respiratory Therapists, Nurses, Technicians involved with CPR
Perrault et al. (2024) (16)	Digital Badge	Physician
Lee-Chavarria et al. (2023) (17)	Digital Badge Microcredential	Clinical Research Professionals
Mashford-Pringle et al. (2023) (21)	Microcredential	Public Health Professionals
Lok et al. (2022) (1)	Microcredential	Pharmacists
Marra et al. (2022) (4)	Microcredential	Pharmacists

*Terms used in the respective article, found through data extraction.

**Table 2:** Summary of characteristics across articles.

Discipline	Number of articles	Percent
Nursing	5	45%
Medicine	4	36%
Pharmacy	4	36%
Clinical Research	3	27%
Other (e.g., respiratory therapist, public health, personal care)	3	27%

Year	Number of articles	Percent
2017	1	9%
2018	0	0%
2019	1	9%
2020	1	9%
2021	0	0%
2022	2	18%
2023	3	27%
2024	3	27%

Type of credential	Number of articles	Percent
Microcredential	4*	36%
Digital Badge	5*	23%
Microlearning for CPD Credit	2	18%
Certificate of Completion	1	9%

Focus of Credential/Badge	Number of articles	Percent
Clinical Research Basics	2	18%
General (focus on perceptions of pharmacy microcredentials)	2	18%
Antimicrobial Stewardship (AMS)	1	9%
Patient Navigation/Navigator Role	1	9%
Indigenous Cultural Safety	1	9%
Bladder Cancer Management (Urothelial Cancer)	1	9%
Working with Dementia Patients in the Evening	1	9%
CPR Performance	1	9%
LGBTQ+ Safe Zones	1	9%

Structure of the learning	Number of articles	Percent
Asynchronous	8	73%
Online activities	8	73%
In-person activities	1	9%
Not Applicable**	3	27%

Requirement to receive credential	Number of articles	Percent
Participatory	3	27%
Competency	4	36%
Unclear	1	9%
Neither	3	27%

*One article called the type of credential a “digital badge microcredential.” **Not a training, but a study of perceptions of microcredentials.

The health profession most commonly discussed in the articles was nursing (*n* = 5, 45%). Pharmacy and general medicine were each included in four (37%) articles while clinical research was included in three (27%). Three articles (27%) related to other health professions such as public health professionals, respiratory therapists, or personal care assistants. About a third of articles incorporated multiple professions (*n* = 4, 36%) while others focused on one profession (*n* = 7, 64%). All the articles included in the review were published within 10 years of the search, with eight of the 11 articles (73%) published after 2021.

Of the 11 articles, two (18%) focused on health care professionals’ perceptions of microcredentials and their use in CPD. One article (9%) described patients’ perceptions of a specific digital badge a provider had on their professional profile. Two of the 11 articles (18%) focused on clinical research basics for the microcredential or digital badge. Two of the 11 articles (18%) discussed unspecified content areas within pharmacy since their main focus was on perceptions of microcredentials. The rest (*n* = 7, 64%) instructed learners on unique, specific topics (e.g., bladder cancer management).

Of the 11 articles, eight (73%) were focused on learning outcomes from specific programs that provided participants with a microcredential, CPD credit, or digital badge. Those eight articles all involved asynchronous and online activities as part of the requirements to receive the microcredential, digital badge, or CPD credit. One of those eight articles, one also included an in-person activity that was needed for measuring skill performance (i.e., Laerdal ResusciAnne and ResusciBaby simulators) (14). Of those eight, four (36%) required competency demonstration through either assessments of knowledge or demonstration of skill; three (27%) were participatory in nature, awarding credit for completion of activities without demonstration of knowledge or skill mastery; and one (9%) was not clear whether credit was awarded by task completion or via a competency-based demonstration. The remaining three (27%) were focused on professional or patient perceptions of microcredentials.

Across these papers, the terms microcredential and digital badge were used to represent the same idea. Noyes and colleagues discussed how a microcredential represented the cumulative completion of a learning while a digital badge indicated progress toward the final completion of learning (13). However, in other articles, a digital badge indicated cumulative completion of a training, such as clinical research basics or LGBT+ safe zone training (15, 16). One article even referred to what the participants gained at the end of the training as a “digital badge microcredential” (17). The use of terminology for the credit received was inconsistent across articles.

In consideration of effectiveness of the microcredentials in this review, the outcomes were variably measured. Two articles focused on professional perceptions of microcredentials indicated a favorable outlook on their possibilities (1, 17). A study of patient perceptions indicated that patients were more likely to trust healthcare providers with the LGBTQ+ digital badge (16). Four studies measured participant perceptions of the knowledge gained from the microcredentials and all indicated increased confidence in their understanding of the designated content (17–20). One of those four also included assessment scores and there was a significant increase in scores throughout the program (18). One study only used assessment scores as indicators of understanding and did not have a statistically significant difference in the measures they used to indicate CPR mastery (14). One study looked at average scores on assessments at the end of each module as well as completion rates (21). The average score was 70% on the end of module assessments for that study (21). Two studies were evaluating the difficulty of their material. One through compliance with rubric standards and the other in relation to a difficulty index (15, 22). Indicators of microcredential effectiveness varied across the articles in this review.

## 4 Discussion

Microcredentials are an emerging approach to learning in the health professions that warrant further consideration as an effective and efficient tool for workforce development. Of note, this review suggests that microcredentials are widely applicable across professions, useful for a wide-range of medical topics, and flexible enough to accommodate various learning modalities. This aligns with Tamouliune et al. who emphasized the flexible nature of microcredentials (11). Hunt et al. also emphasized personalization in CPD with microcredentials, allowing employees to properly prepare for the evolving needs of the workforce across professions (6). However, none of the articles made connection to the possibilities artificial intelligence could provide with microcredentials. There also is current inconsistency in the use of terminology for these professional developments across articles in this review.

The majority of the articles in this review involved professions with established licensing education standards (e.g., nursing), which suggests that microcredentials add value beyond traditional continuing education credits required for licensure. As such, researchers and educators should give consideration to the various ways in which microcredentials might be leveraged to support the development of health professionals beyond traditional training mechanisms. In K-12 education, for example, teachers can earn microcredentials as an “alternative pathway for licensure renewal” [(6), p. 34]. For health professions with existing licensure qualifications, microcredentials may allow for licensed professionals to gain specialized knowledge in emerging areas or niche skills that may not be covered in traditional degree programs or continuing education (23). By earning microcredentials in specific areas, licensed professionals are also able to demonstrate their expertise and readiness for career progression, often in a more flexible and time-efficient option compared to traditional degree programs or continuing education (10, 24).

In professions without licensing requirements, such as clinical research, microcredentials enable the workforce to enhance their skills and signal their capabilities to employers, as demonstrated by the clinical research microcredentials in this review (7, 15). This provides a distinct advantage to webinars or in-person seminars, which do not always signal outwardly the competencies of the learner. By earning multiple microcredentials, health professionals in non-licensed roles can build a portfolio of skills that may lead to new career paths or specializations within health care. As microcredentials continue to gain traction, they also have the potential to serve as a form of industry-recognized qualification, potentially filling gaps where formal licensure does not exist (10, 25).

Similar to the variety of professions using microcredentials, the microcredential topics identified in this review were diverse, including working with dementia patients in the evenings and Indigenous cultural safety. This suggests that microcredentials are versatile and suitable for a diverse range of topics focused on showcasing a specific competency or demonstrating a continued commitment to education. They are also typically low stakes and affordable, allowing learners the opportunity to explore new skills in a manner that will not negatively affect their career or reputation (3, 11).

The papers reviewed in this study described the use of online and asynchronous microcredentials, demonstrating their flexibility in implementation. This flexibility is what makes the microcredentials so attractive to learners, who can then upskill or reskill in a manner that fits their lifestyle or career aspirations (3, 10, 26). For any topic that does not require in person attendance, online and asynchronous microcredentials are a possible method of demonstrating one’s skills to employers (3). A hybrid approach, while not currently discussed in the literature, could be utilized for practical skills that require an in-person assessment. Practical skills, such as first aid or safety-related skills (in a chem lab for example), could be assessed in person while having an online asynchronous component.

Interestingly, the use of artificial intelligence (AI) did not come up in this review, even though it can be supportive of personalized health professional education (27). AI may enhance the effectiveness of microcredentials by tailoring content and learning pathways to individual learners’ needs and preferences, potentially improving completion rates and skill acquisition. AI-powered assessment tools could streamline the evaluation process for microcredentials, allowing for more efficient and scalable credentialing programs (25, 27). AI algorithms even have the potential to analyze job market trends and individual learner profiles to recommend relevant microcredentials for the learner, ensuring aligning between workforce needs and skill development (25). However, this integration raises important considerations, including data privacy concerns regarding the collection and protection of learner information, potential for algorithmic bias in recommendations or assessments, and the need to maintain quality standards for the credential. Striking a balance between leveraging AI’s benefits and addressing these challenges will be crucial for the effective implementation of AI in microcredentials (25, 28).

The language used to describe the microcredentials in this review was inconsistent, a finding consistent with reviews of microcredentials beyond health professions (3). The lack of consistency in terminology puts the validity of microcredentials at risk. With clearly defined characteristics and a framework, the distinction between microcredentials and digital badges can be more easily understood by employers as well as learners. Without a clear understanding, microcredentials could fade out like an educational fad rather than having long lasting impact that could enhance CPD for many professions. The lack of consistent measures of effectiveness also makes it difficult to understand the overall value of the various microcredentials. To address this issue, collaboration is needed to develop a consistent taxonomy and definitions for various types of microcredentials (29). Microcredential providers should clearly articulate the specific competencies, assessment methods, and value of their offerings, while efforts to align microcredential terminology with established qualification frameworks could improve understanding and recognition across organizations and employers (10). Microcredentials could end up going the way massive open online courses (MOOCs) have, where they were initially lauded as the next educational wave but have largely fallen out of favor in part due largely to the lack of understanding between higher education and industry (10, 30). If industry and higher education can reach agreement about terminology and generate market demand, microcredentials would likely experience continued success and growth. For now, microcredentials aspire to demonstrate a person’s commitment to learning and professional development, however more work is need to optimize the emerging approach to workforce development.

## 5 Limitations

Due to the rapid nature of this review, it is possible that literature was missed in the searches. New literature could have also been published on the topic of microcredentials since the database searches were conducted. This review was also limited to articles published in English which could have limited the research found from non-English-speaking countries. This review focused on peer-reviewed studies of microcredentials; however microcredential hosting platforms, such as Credly and Accredible, could further inform understanding of types of microcredentials being offered to health care professionals. Further, academic credit from microcredentials was not considered in this review, due to the exclusion criteria, but would also be another avenue of focus for future understanding of microcredentials.

## 6 Conclusion

Microcredentials provide promising opportunities for versatile continuing professional development in health professions. Many are optimistic about the possibilities for reskilling and upskilling in a variety of topics across multiple professions. However, the need for consistent terminology and consistent views of their market value are prominent challenges in the effective implementation of microcredentials on a broader scale.
