# Supplementary material for: A review of microcredentials in health professions continuing professional development
Source: Front Med (Lausanne). 2025 Feb 3;12:1532811. doi: 10.3389/fmed.2025.1532811 (PMC11841466; doi:10.3389/fmed.2025.1532811)
Supplement: Supplementary file 1 [file Table_1.DOCX]

Supplementary Material

# Supplementary Material – Appendix A: Codebook for Data Extraction

| Code | Subcode | Description | Select Examples: from Romero-Clarà et al. (2024) |
| --- | --- | --- | --- |
| Characteristics | Profession | What health care profession is being focused on in this study? | Nurses, Physicians, Researchers, Pharmacists (p.5) |
|  | Topic | Content Area of Focus What knowledge and/or skills are addressed? | Treatment of Urothelial Carcinoma (p.3) |
|  | Setting of training | Virtual (synchronous/asynchronous); in-person (health care facility/institution of higher education) | The “E-learning Pills on Immunotherapy in Urothelial Cancer ” (E-PIMUC) project (Hosted on www.e-oncologia.org) (p.2) |
|  | Structure of the training | Modules, skill based practice, micro-learning videos/questions, etc. | digestible units called learning pills. The average learning pill duration is 15 min and the longest is 45 min. (p. 3) |
|  | Type of credit/credential/badge | What is the credit for? Is it a continuing education credit to support licensing? Is it a digital badge to signal something? Is it a micro-credential for a portfolio? | The program was worth 2.4 professional credits. (p.2) |
|  | Study Design | Was the study quantitative, qualitative, quasi-experimental, etc.? Was this a survey, observational study?  What was being measured or evaluated? | Our mixed methods approach comprised a single group, pre/post study with a combination of quantitative and qualitative evaluation instruments and methods (p.3) |
| Outcome |  | What were the outcomes found in the study? Survey results, learning growth, etc. | Satisfaction with the course was rated as Remarkable (4) by 37.8% of participants and Excellent (5) by 43.1%, on a Likert scale of 1 to 5 (p.6) |
| Implications |  | Potential impact of the study What do the findings from the study mean for the broader field? | E-PIMUC attracted a significant number of nursing professionals. This is promising, since nurses play a pivotal role not only in administering treatments but in educating patients and managing adverse effects related to new cancer treatments, including immunotherapy. (p.8) |
| Challenges or Limitations |  | What were the limiting factors in the study? What challenges arose with the study/credential? | The main limitations of our study include the reliance on self-reported rather than objective assessments, and the lack of long-term benefit reporting or impact assessment, with evaluations limited to relatively short-term outcomes (p.9) |
| Other |  | Anything notable from the articles, reflections from the articles, etc. |  |

**Supplementary Material:** Codebook for Data Extraction
